# Case discussions of missed traumatic fractures on computed tomography scans

**DOI:** 10.4102/sajr.v26i1.2516

**Published:** 2022-11-30

**Authors:** Amy J. Spies, Maryna Steyn, Desiré Brits, Daniel N. Prince

**Affiliations:** 1 Human Variation and Identification Research Unit (HVIRU), School of Anatomical Sciences, Faculty of Health Sciences, University of the Witwatersrand, Johannesburg, South Africa; 2 Department of Basic Medical Sciences, University of Arizona College of Medicine, Phoenix, Arizona, United States of America; 3 Department of Diagnostic Radiology, Faculty of Health Sciences, University of the Witwatersrand, Johannesburg, South Africa; 4 Department of Radiology, Wits Donald Gordon Medical Centre, Johannesburg, South Africa

**Keywords:** radiology, diagnostic errors, fracture misdiagnoses, traumatic fractures, emergency department

## Abstract

Radiological diagnostic errors are common and may have severe consequences. Understanding these errors and their possible causes is crucial for optimising patient care and improving radiological training. Recent postmortem studies using an animal model highlighted the difficulties associated with accurate fracture diagnosis using radiological imaging. The present study aimed to highlight the fact that certain fractures are easily missed on CT scans in a clinical setting and that caution is advised. A few such cases were discussed to raise the level of suspicion to prevent similar diagnostic errors in future cases. Records of adult patients from the radiological department at an academic hospital in South Africa were retrospectively reviewed. Case studies were selected by identifying records of patients between January and June 2021 where traumatic fractures were missed during initial imaging interpretation but later detected during secondary analysis or on follow-up scans. Seven cases were identified, and the possible causes of the diagnostic errors were evaluated by reviewing the history of each case, level of experience of each reporting radiologist, scan quality and time of day that initial imaging interpretation of each scan was performed. The causes were multifactorial, potentially including a lack of experience, fatigue, heavy workloads or inadequate training of the initial reporting radiologist. Identifying these causes, openly discussing them and providing additional training for radiologists may aid in reducing these errors.

**Contribution:**

This article aimed to use case examples of missed injuries on CT scanning of patients in a South African emergency trauma setting in order to highlight and provide insight into common errors in scan interpretation, their causes and possible means of mitigating them.

## Introduction

Traumatic fractures are frequently encountered in both postmortem and clinical settings.^1,2^ Investigating these cases and providing appropriate patient care and management rely on correctly diagnosing these fractures.^3,4^

**FIGURE 1:**
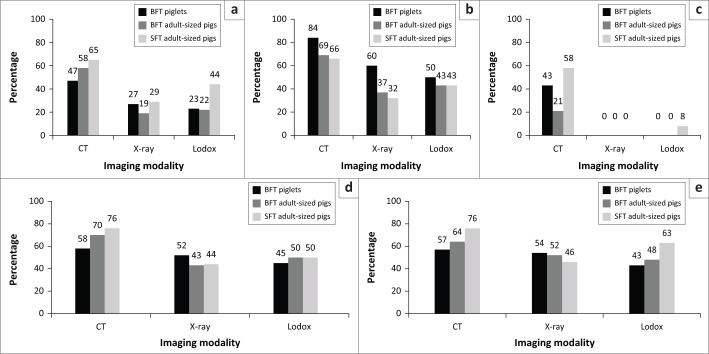
Comparison of percentage of blunt (BFT) and sharp force (SFT) lesions detected by CT, X-ray and Lodox in various body regions in a postmortem context using a pig model. Piglets (black bars) were included to simulate cases of child abuse. (a) Skull, (b) ribs, (c) vertebrae, (d) forelimbs, (e) hindlimbs. Percentages taken from Spies et al.^5,6,7,8^

Recent studies^5,6,7,8,9^ using an animal model have found that in postmortem radiological assessments of blunt and sharp force trauma, CT, although more sensitive than X-ray and low-dose full-body X-ray (Lodox), fails to detect between 16.0% and 79.0% of bone lesions, with all modalities commonly missing skull and vertebral lesions (Figure 1). It was recommended that in postmortem cases, osteological examinations of these regions be performed if trauma is suspected, rather than simply relying on radiological imaging.^5,6,7,8,9^ The potential reasons for these missed lesions in the postmortem context range from superimposition of structures on radiographs to a lack of observer experience and training.^5,6,7,8,9^

While the radiological analysis in postmortem and clinical settings may differ, the potential reasons for failing to detect traumatic fractures may largely be the same. In a clinical setting, major detection errors may result in inadequate patient care and management and could have life-threatening results.^3,4^ However, radiology is between the sixth and eighth most frequent recipient of malpractice claims, with up to 60.0% of claims citing failure to diagnose either soft tissue or skeletal abnormalities.^10,11,12,13^ The risk of failing to detect fractures needs to be limited as much as possible, which can be achieved by recognising fractures commonly missed during initial radiological assessments, and potential reasons for these errors.^3,4,10^

While the potential reasons for missing skeletal lesions radiologically in a postmortem context have been explored,^5,6,7,8,9^ this study aims to evaluate this in a clinical context. The purpose of this study was to retrospectively review patient records at an emergency radiological department in South Africa and analyse specific cases to demonstrate the errors with regard to the radiological interpretation of traumatic fractures.

## Materials and methods

Records of adult patients (18–99 years) who presented to the radiological department at an academic hospital in South Africa, over a 6-month period between January 2021 and June 2021, were retrospectively reviewed by the senior author (D.N.P.). In this department, initial imaging interpretation is performed by the trainee radiologist (registrar) on call, and these initial reports are checked by a consultant radiologist during the following shift. Junior and senior registrars typically have less than and more than two years of experience prequalification, respectively. Junior and senior consultants, respectively, typically have less than and more than five years of training post qualification. Fractures detected during both the initial and secondary imaging interpretations are recorded.

Case studies were selected from patient records that showed fractures not diagnosed upon initial interpretation but identified during secondary analysis of the initial scan or upon analysis of a follow-up scan. Patient age, sex, case history, time of day initial imaging was performed, and the level of experience of the registrars and consultants were recorded. No patient identifying information was recorded, and patient anonymity was ensured throughout the study.

### Case reports

Seven cases with fractures not diagnosed at the initial interpretation of the radiological images, but identified during secondary analysis, were selected. These cases may not represent all of those where fractures were not diagnosed but were selected as they each had specific teaching points that could be used to highlight learning opportunities. They all also demonstrate specific potential pitfalls in detection and interpretation.

#### Case 1

**FIGURE 2:**
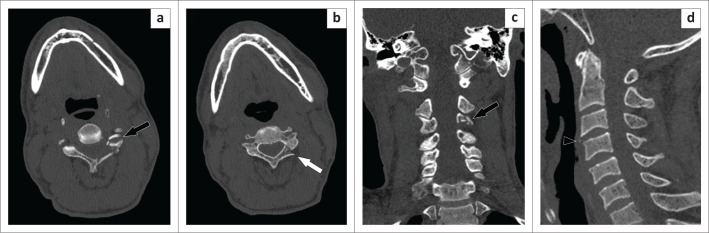
Axial (a and b) and coronal (c) reconstructions of the cervical spine CT showing comminuted fractures of the left superior and inferior facets of C4 (black arrow) and left lamina of C4 (white arrow). Sagittal (d) reconstruction shows a small avulsion fracture of the anterosuperior margin of the C4 vertebral body (black arrow). These fractures represent an unstable cervical spine injury.

A 57-year-old man presented to the emergency department (ED) following a motor vehicle accident (MVA) and had been stabilised with a cervical collar. The patient experienced a loss of consciousness, headache and cervical spine tenderness. A head and cervical spine CT was performed at 03:23 on a Saturday. Initial interpretation by a junior registrar failed to detect any fractures and the cervical collar was removed. Soon thereafter, the patient developed ‘unexplained quadriplegia’. A subsequent CT pan-scan was performed at 10:00 the same day to search for any thoracolumbar spine fractures to explain the symptoms. A junior consultant identified a fracture of the C4 vertebra on the CT pan-scan, which, in retrospect, was visible on the initial CT (Figure 2a–d). Had the findings been detected upon initial interpretation and relayed to the referring clinicians, the cervical spine collar would not have been removed and this outcome may have been avoided.

#### Case 2

**FIGURE 3:**
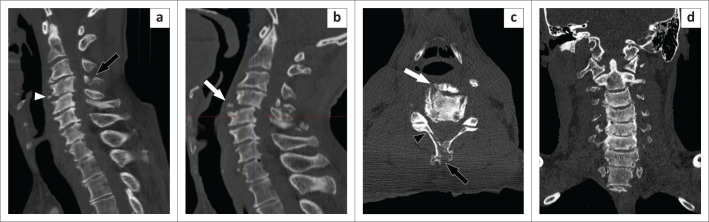
Sagittal reconstruction of the initial CT of the cervical spine (a) showing the C4 spinous process fracture (black arrow). The follow-up CT (b) shows worsened retrolisthesis of the C4 on C5 vertebra with an anterior teardrop fracture of the C4 vertebra (white arrow). This fracture is visible in (a) but was called a ‘fractured osteophyte’ (white arrowhead). The axial (c) and coronal (d) reconstructions of the scan show how the degenerative cervical spine changes may make diagnosing fractures challenging – the teardrop fracture is pointed out by the white arrow in (c) and may have been misinterpreted as an osteophyte (white arrowhead). The black arrowhead in (c) demonstrates a right lamina fracture, and the black arrow again points out the spinous process fracture.

A 60-year-old man presented to the ED following a MVA. The patient had an altered level of consciousness, headache, cervical spine tenderness and was unable to move his legs. A head and cervical spine CT was performed at 17:56 on a Monday. The junior registrar noted severe degenerative changes in the cervical spine but no fractures. Upon review, the senior consultant detected a missed C4 spinous process fracture – a stable injury – but no other abnormalities (Figure 3a). A follow-up CT pan-scan was requested to confirm that there were no thoracolumbar spine fractures that could have explained the patient’s worsening neurological symptoms, and a different consultant radiologist detected retrolisthesis of C4 on C5 (Figure 3b–d). Secondary interpretation of the initial CT was performed by this same consultant, who noted an anterior teardrop fracture of the C4 vertebra (Figure 3b–d), making the cervical spine injury unstable.

#### Case 3

**FIGURE 4:**
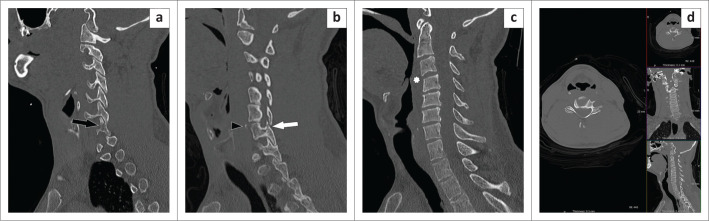
Paramidline sagittal (a and b) reconstructions of a cervical spine CT showing a fracture of the right inferior facet of the C6 vertebra (black arrow), a fracture of the right superior facet of the C7 vertebra (white arrow) and a fracture of the anterosuperior margin of the body of C7 (black arrowhead). A midline sagittal reconstruction (c) demonstrates reversed cervical spine lordosis but no listhesis. Associated prevertebral soft tissue swelling is present (white asterisk). Note that the patient was ‘scanned skew’ (d).

A 34-year-old man involved in a MVA, presented to the ED with loss of consciousness, a large head wound, cervical spine tenderness, a fractured left femur and rib fractures. A CT pan-scan was performed at 17:47 on a Saturday. A senior registrar performed the initial interpretation, reporting the cervical spine CT as normal. A junior consultant then reviewed the CT and noted several vertebral fractures that were missed initially (Figure 4a–d). Associated prevertebral soft tissue swelling was also noted (Figure 4a–d).

#### Case 4

**FIGURE 5:**
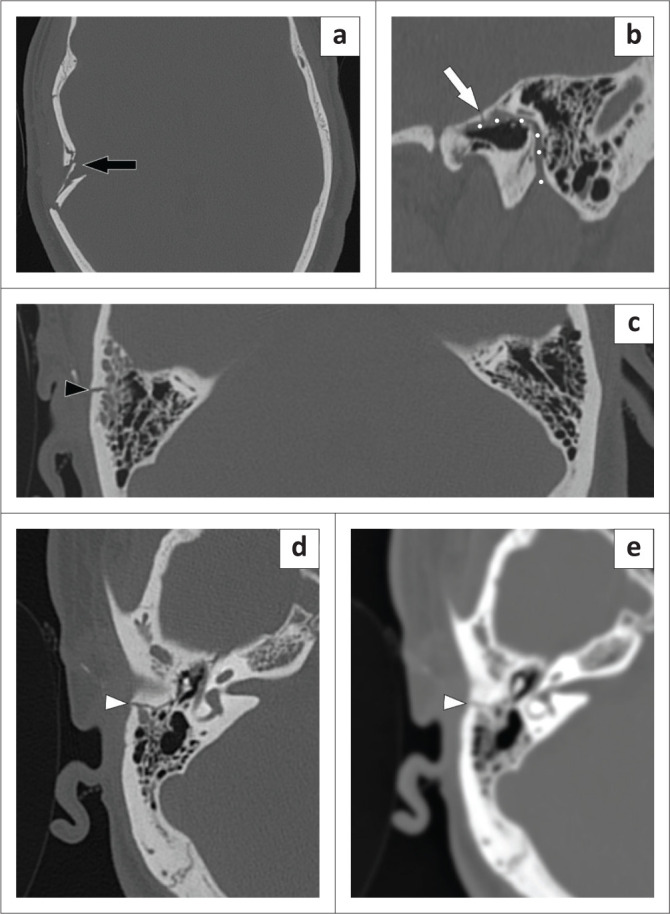
Axial CT of the head (a) demonstrating the depressed right squamous temporal bone fracture (black arrow) detected on initial interpretation. An oblique reconstruction of the right temporal bone (b) shows that the fracture (white arrow) extends into the facial nerve canal (highlighted here by the white dots). Specifically, it involves the tympanic segment of the canal. The fracture line extending into the mastoid part of the temporal bone is pointed out by the black arrowhead in the axial image (c) – note the fluid in the mastoid air cells and compare it to the well-aerated left mastoid air cell; a secondary sign of temporal bone fracture. The fracture line (white arrowhead) is much more conspicuous on the axial cut of the bone reconstruction algorithm (d) than the soft tissue algorithm (e), despite both being set to a standard ‘bone window’.

A 29-year-old man sustained blunt trauma to the head and a CT on the day of injury demonstrated a large, depressed skull fracture which was treated conservatively. Ten days later, a high-resolution temporal bone CT was performed at 12:27 on a Wednesday after the patient complained of a 1-day history of right facial nerve fallout. Initial interpretation by a junior registrar noted the depressed squamous temporal fracture (Figure 5a) but failed to notice that it extended into the petrous and mastoid regions to involve the facial nerve canal within the otic capsule, known as an ‘otic capsule-violating’ fracture (Figure 5b–e).

#### Case 5

**FIGURE 6:**
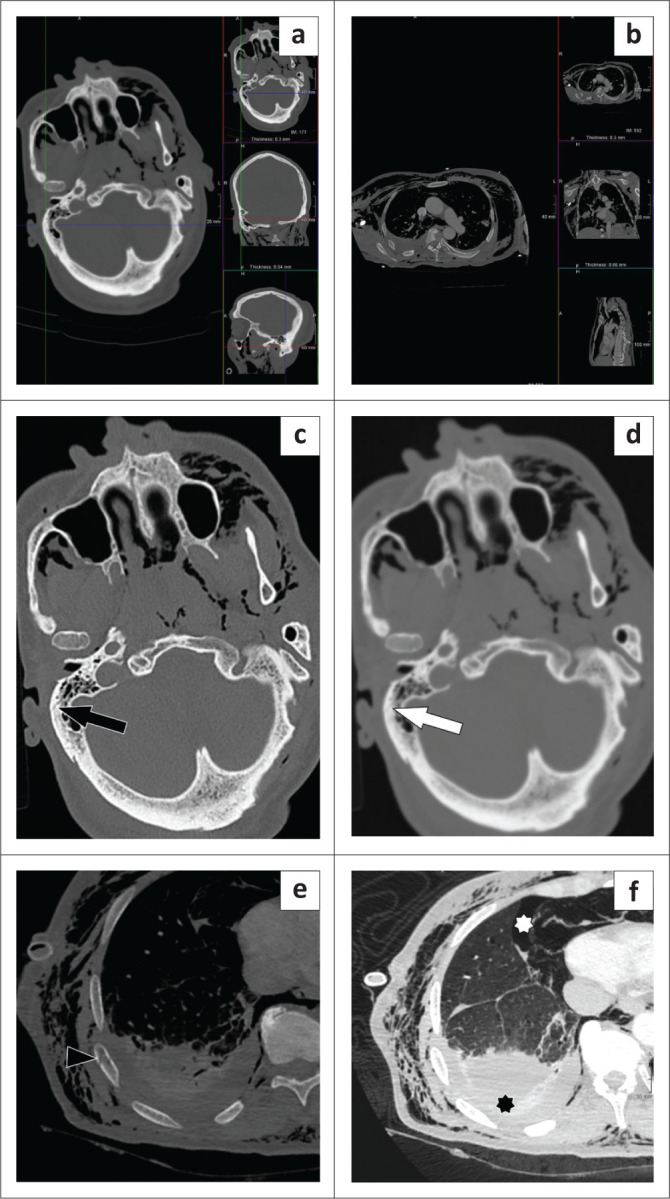
A CT pan-scan showing extensive subcutaneous emphysema of both the head (a) and chest (b). When viewed without straightening the scan using multiplanar reconstruction (c), the right temporal bone otic capsule–sparing fracture is not easy to detect even utilising the bone reconstruction algorithm (black arrow) and nearly invisible (white arrow) on the soft tissue reconstruction algorithm (d). The bone window (e) demonstrates an initially overlooked nondisplaced right 9th rib fracture (black arrowhead). Note the right haemothorax (black star) and pneumothorax (white star) seen on the lung window of the chest CT (f). Also note that the patient was ‘scanned skew’.

A 65-year-old man with an unknown mechanism of trauma presented with a right pneumothorax and extensive subcutaneous emphysema. A CT pan-scan (Figure 6a–f) was performed at 12:48 on the Tuesday of presentation and was initially reported as normal by a junior registrar. However, on secondary analysis by a consultant, several missed fractures were detected, including an otic capsule–sparing right temporal fracture (Figure 6c–d) and multiple nondisplaced right rib fractures (Figure 6e–f).

#### Case 6

**FIGURE 7:**
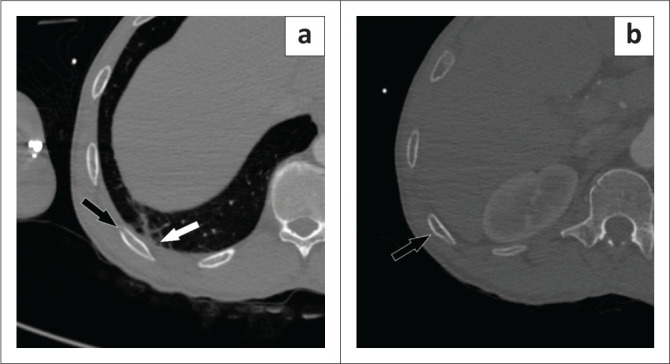
Axial bone reconstruction of a CT (a and b) of the chest demonstrating subtle buckle-type fractures of the 10th and 11th ribs (black arrows) associated with a small lung contusion (white arrow).

An adult man of unknown age, involved in a MVA, presented to the ED on a Sunday. The patient had a head injury and a fractured tibia and fibula confirmed at radiography. A CT pan-scan was performed at 12:42, and initial interpretation by a junior registrar failed to diagnose subtle fractures of the right 10th, 11th and 12th ribs with associated lung contusions (Figure 7a–b). These were identified by a junior consultant during secondary interpretation. No pneumothorax was associated with these fractures.

#### Case 7

**FIGURE 8:**
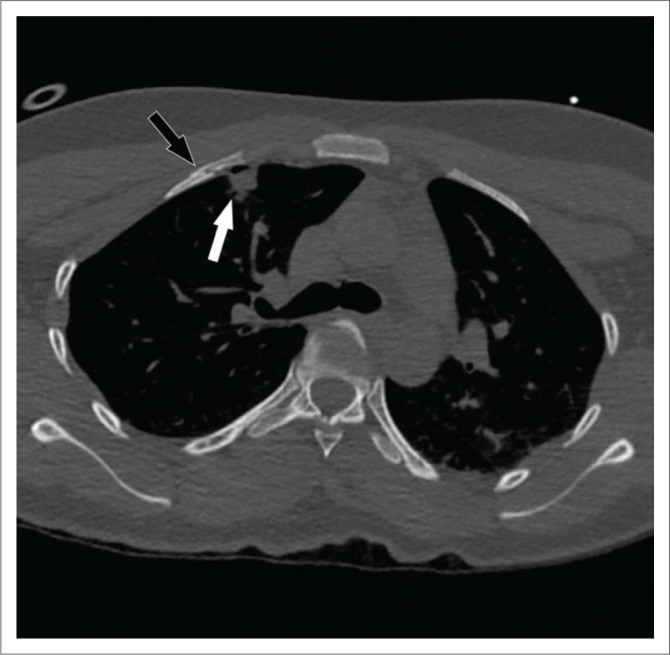
Axial bone reconstruction CT of a the chest demonstrating a minimally displaced second rib fracture (black arrow) associated with a small lung laceration and contusion (white arrow). As with cases 5 and 6, lung injuries such as contusions or lacerations should prompt careful search for associated rib fractures in the trauma patient.

A 30-year-old man involved in a MVA had a loss of consciousness with an open midshaft fracture of the tibia and fibula confirmed on plain radiographs. A junior registrar performed the initial interpretation of the CT pan-scan at 02:05 on a Monday, reporting no additional fractures. Secondary analysis by a junior consultant, however, revealed a nondisplaced fracture of the right second rib and a buckle-type fracture of the right third rib (Figure 8). While small lung contusions and a laceration were associated with these fractures, there was no pneumothorax (Figure 8).

## Discussion

A growing body of literature shows that there are many radiological misdiagnoses of fractures of the ribs, vertebrae and cranial region, both in clinical and postmortem contexts,^5,6,7,8,9,12,13,14^ and these diagnostic errors could have severe consequences. A review of trauma patients who had undergone CT scanning at an academic hospital in South Africa similarly revealed that many of the fractures not detected on initial CT imaging interpretations are those of the vertebrae, temporal bone and ribs. These diagnostic errors resulted in a range of consequences, from pain and discomfort due to undiagnosed subtle rib fractures (cases 5, 6 and 7) to spinal cord pathology due to undiagnosed cervical spine fractures (cases 1 and 2). Had these fractures been diagnosed initially, surgical plans and patient management strategies would likely have been altered.

While some of the fractures missed in the present study are subtle (cases 5, 6 and 7), others are very evident on the initial scans (cases 1, 2 and 4). That these fractures were not detected or were misinterpreted as other abnormalities are likely due to a combination of factors termed ‘observer errors’ or other extenuating circumstances. It could even suggest inadequate radiology training and/or inadequate image interpretation techniques.^3,4,10,15,16^

Three types of observer error have been described.^3,4,16^ Scanning error occurs when the observer fails to fixate on the region where the fracture is present. Recognition error occurs when the observer fixates on the correct region where the fracture is present but fails to identify it. Decision-making error occurs when the observer correctly detects the abnormality but misinterprets it as something else.

In a postmortem setting, missed fractures were shown to be partially due to a lack of radiological experience and training.^9^ Similarly, in a clinical setting, all three types of observer error may occur as a result of the level of experience and training of the reporting radiologist.^3,4^ All but one of the initial imaging interpretations of the cases were performed by junior registrars with less than two years of training. This lack of experience may result in junior registrars failing to identify fractures and secondary signs thereof. For example, in cases 5, 6 and 7, a pneumothorax (case 5) and small lung contusions and a soft tissue laceration (cases 6 and 7) were associated with undiagnosed rib fractures. Moreover, the presence of subcutaneous emphysema adjacent to the temporal bone and fluid within the mastoid air cells (case 5) or the presence of prevertebral soft tissue swelling in the cervical region (case 3) should have prompted the initial reader to look carefully for fractures in these regions. This was also found to be the case in postmortem examinations of sharp force trauma, where the presence of soft tissue lesions enabled forensic anthropologists to detect underlying osseous trauma.^8^ Adequate training and experience would likely result in the radiologist identifying these ‘secondary signs’ of fractures and then actively searching for and detecting any associated osteological trauma.

Lack of experience with and knowledge of normal skeletal anatomy and skeletal growth, development and degeneration may result in misdiagnosing fractures as normal anatomical variants or other pathological conditions.^3,17^ In case 2, recognition and decision-making errors were made as cervical spine fractures were mistaken for degenerative changes. Increased osteological experience and knowledge would potentially reduce errors, as recollection of previously encountered pathologies and anatomical variants, as well as a knowledge of those not yet encountered, could improve recognition rates.^3^

However, diagnostic errors are also commonly made by more experienced radiologists. The cervical spine fractures in case 2 were also initially misdiagnosed by a consultant as a stable injury, having only detected the spinous process fracture and missing the unstable teardrop fracture (recognition error). One potential reason for this could be that as experience level increases, the speed with which the observer interprets images increases, and as a result, so does the number of detection errors.^3,4,18^

Errors of speed may also occur due to increased workloads and a reduction in the time available for radiological reporting.^3,10,18^ The number of radiological examinations performed has increased exponentially over the years, which reduces the amount of time a radiologist can dedicate both to interpreting these images and to continued education and training.^10,18^

Additionally, error rates may be related to the level of alertness of the radiologist.^3,19^ Only two scans (cases 4 and 5) were performed during the institution’s ‘normal business hours’, while initial imaging of cases 1 and 7 were performed during overnight shifts (at 03:23 and 02:05, respectively), when it is likely that the radiologist on call is tired, resulting in a reduced level of alertness and consequently an increased error rate.^3,19^ Initial interpretations of the remaining three cases (2, 3 and 6) were performed after-hours during weekends and in the early evening. Busy days with high workloads may also result in fatigue and, as a result, higher error rates.^3,19^

Another type of diagnostic error is a satisfaction of search error, which is the result of one abnormality causing the attention of the radiologist to be diverted away from another, such as a fracture, resulting in this abnormality being overlooked.^3,4,16^ In case 5, the extensive surgical emphysema may have distracted the reporting registrar, diverting their attention away from the more subtle rib fractures. The senior consultant reviewing case 2 may have also fallen into this trap in that they diagnosed the spinous process fracture and thereafter was ‘satisfied’ that an abnormality was detected to explain the patient’s symptoms and simply moved on to another search area.

In cases of polytrauma, CT pan-scans are often requested and include noncontrast head, cervical spine, contrast-enhanced chest and multiphase abdomen and pelvis imaging. Occasionally, these scans will also include peripheral or cervical CT angiograms, if clinically indicated. Trauma patients, for various reasons, are sometimes placed on the CT table in ways that result in the scans not being true axial cuts such as in cases 3 and 5. The resultant asymmetry when the patient is ‘scanned skew’ can make fractures of the skull and spine difficult to detect (cases 3 and 5). This was also noted in the postmortem studies using pig models, where some of the X-ray and Lodox images were ‘skew’, and some scans were not true-lateral or true-frontal images.^5,6,7,8,9^ Viewing the scan of each body region in multiple planes and in three-dimensional volumetric reconstructions is therefore essential. However, this adds drastically to the number of images to be reported. Furthermore, different image reconstruction algorithms – that is, soft tissue and bone reconstructions, which, if reviewed with special care, may have resulted in the temporal bone fractures in cases 4 and 5 being detected more easily – essentially double the number of images per body region. All of these image acquisition and postprocessing techniques result in a very large dataset to read and interpret. It is therefore not sufficient to simply view the images as the patient is being scanned but is crucial that radiologists carefully and systematically view all body regions in different planes to ensure that all injuries are diagnosed.

As in the postmortem contexts,^5,6,7,9^ there are many potential reasons for radiologists failing to detect fractures in a clinical setting, and these often exist in combination.^17,19^ Diagnostic errors can potentially be limited by improvements in knowledge, training and experience, as well as double reporting and improved communication between radiologists and clinicians.^3,4,10,12,13,16^ In addition, having open discussions about errors and their causes could aid in reducing errors.^3,4^ One way of addressing this, which was trialled by the senior author (D.N.P.), is to institute ‘radiology morbidity and mortality meetings’ (‘M&Ms’) where cases such as those discussed in this study are presented monthly. These were conducted as non-confrontational, non-accusatory tutorial sessions in which potential causes for the misdiagnoses were hypothesised and learning points from each case were emphasised. This was met with positive feedback from the registrars and could be a powerful training tool if routine ‘M&Ms’ are held in academic radiology departments. Cases in these ‘M&Ms’ were anonymised, and the interpreting radiologists were never named so as not to place blame or judgement but to create learning opportunities in order to reduce diagnostic errors and improve patient care and management.^3,4,16^

## Conclusion

Like the imaging of trauma in postmortem contexts, the causes of diagnostic errors in detecting traumatic fractures in a radiology department are multifactorial and may include lack of radiologist training, knowledge and experience; fatigue and heavy workloads; and inadequate image interpretation techniques. Understanding these errors and their root causes is crucial to improving the efficacy of radiological departments. Additional training and open discussions of these errors and their causes, treating them as learning opportunities, can aid in reducing the prevalence of reporting errors. In particular, a high index of suspicion is important, especially when injuries to the chest and skull are concerned, as these are the most commonly misdiagnosed regions.
